# Surveillance considerations for malaria elimination

**DOI:** 10.1186/1475-2875-11-304

**Published:** 2012-08-31

**Authors:** Victoria C Barclay, Rachel A Smith, Jill L Findeis

**Affiliations:** 1 Department of Biology and Center for Infectious Disease Dynamics, The Pennsylvania State University, University Park, PA, USA; 2 Department of Communication Arts & Sciences, Human Development & Family Studies, and Center for Infectious Disease Dynamics, The Pennsylvania State University, University Park, PA, USA; 3 Division of Applied Social Sciences (DASS), Agricultural & Applied Economics, CAFNR, University of Missouri, Columbia, MO, USA

**Keywords:** Surveillance, Co-ordination, Data sharing, Data reservoir platforms

## Abstract

Constant malaria monitoring and surveillance systems have been highlighted as critical for malaria elimination. The absence of robust monitoring and surveillance systems able to respond to outbreaks in a timely manner undeniably contributed to the failure of the last global attempt to eradicate malaria. Today, technological advances could allow for rapid detection of focal outbreaks and improved deployment of diagnostic and treatment supplies to areas needing support. However, optimizing diffusion activities (e.g., distributing vector controls and medicines, as well as deploying behaviour change campaigns) requires networks of diverse scholars to monitor, learn, and evaluate data and multiple organizations to coordinate their intervention activities. Surveillance systems that can gather, store and process information, from communities to national levels, in a centralized, widely accessible system will allow tailoring of surveillance and intervention efforts. Different systems and, thus reactions, will be effective in different endemic, geographical or socio-cultural contexts. Investing in carefully designed monitoring technologies, built for a multiple-acter, dynamic system, will help to improve malaria elimination efforts by improving the coordination, timing, coverage, and deployment of malaria technologies.

## Background

For those countries close to malaria elimination, real-time, on-going monitoring systems are important for at least four reasons. They allow for (1) rapid detection of existing, new or re-introduced (e.g, across country and regional borders) infections [1,2]; (2) identification of periods of low transmission (e.g, from symptomatic and asymptomatic infections) when the parasite population could be most amenable to elimination [3,4]; (3) understanding trends in malaria incidence and prevalence (shifts in age groups, increasing parasite heterogeneity, changes in seasonality) and, (4) detection of resistance. As malaria becomes less prevalent in a country, intervention efforts may weaken, which in turn may create more resistant parasite and mosquito populations [5-7]. In addition, surveillance itself can be an intervention that reduces transmission by identifying and rapidly treating infections from the infectious reservoir [8]. Monitoring systems that can rapidly detect and help excise low-level malaria transmission, identify optimal intervention windows and flag emerging resistance are all essential for disease elimination.

## Discussion

A real-time, on-going, integrated data reservoir capturing multiple scales of disease dynamics - from cells to society is a plausible, achievable enterprise for malaria elimination in the next decade. The finest, comprehensive, malaria-monitoring systems include portable and sensitive diagnostic tests, real-time data about patients showing drug resistant parasites and vectors showing insecticide resistance, transmission intensity markers (e.g, sampling, surveys and biomarkers), climatic data (e.g, rainfall as early warning systems), geo-spatial and demographic information for villages known to be particularly at risk, and continuous, frequent (e.g, at least monthly) local-level malaria incidence counts [8-13]. Together, these data represent biological, clinical, social scientific and logistical information that inform different aspects of disease detection and intervention planning aiming to sustainably impact malaria-burdened populations.

However, monitoring is not enough: learning and evaluation are also needed, which is complicated in a multiple-organization (National Malaria Control Programmes (NMCPs), government ministries, international health agencies, private industry, non-governmental organizations, funding agencies, and local health organizations, etc.,) multiple-acter (scientists, politicians, and interventionists) effort [14]. To optimize malaria intervention efforts, both organizations and acters need (a) timely, robust information about disease epidemiology and dissemination activities (e.g, bed nets, spraying, testing supplies, drugs, and vaccine trials); (b) an ability to access data and each other to coordinate activities for integrated vector-control and public health responses; and, (c) conditions (both online and offline) that facilitate data sharing and optimal, group-based decision-making [15]. Research into designing data reservoir platforms that facilitate monitoring, learning and evaluation (MLE) among multiple acters is needed to optimize coordinated, integrated disease detection and intervention efforts.

However, the use and maintenance of MLE systems requires a level of infrastructure (communication networks) absent in some malaria-burdened countries. For example, in some of the most remote parts of Africa, there are few landline telephones, computers with fast internet-access, or roads in good condition, for the rapid transfer of disease information [16,17]. Mobile phones, diffusing widely in African countries, may offer great possibilities for MLE efforts. Mobile phones can allow researchers to collect and share data faster and easier in countries lacking other infrastructures. Data can be collected unobtrusively: monitoring people’s movement back and forth, from low-transmission Zanzibar to malaria-burdened Tanzania [2]. Short message service (SMS) technology has also been utilized to strengthen the routine reporting of anti-malaria drug supplies at health facilities [18,19].

Notably, real-time, comprehensive monitoring does not inherently lead to access or to collaboration and coordination among different organizations and acters. For enriched MLE technologies, such as mobile phones, to improve coordination, optimization, and deployment of intervention resources [2,20], there should be investment in real-time, updating data reservoir platforms that are compatible across hardware systems and national boundaries, readily accessible for scientists and interventionists, and built to facilitate transdisciplinary learning and evaluation. If each entity interested in monitoring gathers, stores, and shares their data differently, by the time integration takes place, the data’s utility may have expired. Likewise, multiple organizations attempting to deploy interventions may duplicate services in some areas while missing others needing assistance, particularly when NCMPs are weakened due to other factors such as post-emergency or during war-time. Further, compatible data management tools should be developed regardless of the specific factor being surveyed (e.g, drug and vector resistance, number of infected cases, climate data, and so forth) because future research efforts may benefit from integrating these different factors to best manage malaria efforts. Last, designing platforms and management tools informed by social science, computer science, biology and epidemiology are needed to help facilitate trans-disciplinary learning, and system-wide collaboration and coordination.

Platforms are already being developed by those who recognize the benefits of integrating global positioning systems (GPS), geographic information systems (GIS) and mobile computing technology into modern reporting applications [11]. Platforms that can collect information on the spatial distribution of malaria and other variables such as weather and climate [21,22], land use and demography [23] and vector breeding sites [24,25] will help to identify malaria risks and distribution across a variety of scales (i.e. globally, nationally and locally). The development of those platforms for mapping malaria risk is encouraging, and could be expanded to include the collection of other disease indicators.

First, MLE systems should be designed with local communities in mind, such as allowing for data entry interfaces and reports to be personalized by the user. Different systems may be more effective in one setting over another. Existing models suggest that in communities where disease incidence is low, a simple ‘eyes and ears’ approach for early treatment - seeking could be equally effective as more technical methods of disease detection [14,26]. Community vigilance is not sufficient to achieve elimination [3], however, because community vigilance at the local level may not inform national-level policies or activities and because the time delay between the recognition of disease symptoms (e.g. fever) and the reporting to a health clinic, extends the window for onward transmission. As technology continues to diffuse across African countries, MLE systems could complement community approaches by increasing the speed by which regional and national surveillance teams are alerted to local events and prepare intervention services for local demands. Analogous to improvements in the marketing of agricultural products in African countries due to rapid access to information on market prices [27], MLE systems for disease surveillance should work to complement, not replace, community vigilance.

As in the development of agricultural innovations and systems [28], end-users and stakeholders must also be involved in the design process. Different countries have implemented their own malaria surveillance systems with varying degrees of success [9,11,29-31]. For new MLE-collaboration platforms to succeed, targeting and tailoring of MLE technologies, standards, and communication to users is needed to improve the comprehension, diffusion and adherence of new surveillance tools [32].

In essence, ensuring access to data within and across different regions and countries is as important as developing and diffusing technologies to monitor malaria dynamics and intervention activities. Data collected and stored in an accessible manner can quicken the speed at which scientists can evaluate an intervention’s impact, forecast possible changes to improve future efforts, and adjust to incoming results. Speed plays an important role in malaria management, where outbreaks occur across numerous ecosystems and throughout the seasonal variation [33]. With current, dynamic, accessible information and trained personnel who can to learn from it and act on it, resources can be allocated more efficiently and adjust programmes and policies more appropriately.

## Conclusions

In summary, malaria MLE systems from community to national levels, and informed reactions (interventions), can provide valuable insights needed to understand, forecast, and evaluate complex, multiple-organization-and-acter efforts, such as eliminating malaria. In order to meet malaria elimination objectives, monitoring systems must be able to respond rapidly to the heterogeneity in malaria epidemiology. Many malaria-burdened countries are experiencing advances in technology and research, which may be harnessed to optimize the feasibility, efficiency and cost-effectiveness of readily accessible, shared data collection, evaluation, and collaboration systems. This research should strive to improve MLE systems in even the most remote locations. Investment in integrated MLE systems alone will not eradicate malaria, but it will bring us closer.

## Abbreviations

MLE: Monitoring learning and evaluation; NMCPs: National malaria control programmes; GPS: Global positioning systems; GIS: Geographic information systems; SMS: Short message service.

## Competing interests

The authors declare that they have no competing interests.

## Authors’ contributions

VB reviewed the literature and drafted the paper. RAS reviewed the literature and edited the manuscript. JFL edited the manuscript. All three authors conceived the idea equally. All authors read and approved the final manuscript.
